# A Systems-Based Analysis of the CardioMEMS HF Sensor for Chronic Heart Failure Management

**DOI:** 10.1155/2019/7979830

**Published:** 2019-07-17

**Authors:** Jeffrey S. Tran, Aaron M. Wolfson, Daniel O'Brien, Omid Yousefian, David M. Shavelle

**Affiliations:** ^1^Department of Internal Medicine, Keck School of Medicine of USC, Los Angeles 90033, CA, USA; ^2^Division of Cardiovascular Medicine, University of Southern California, Los Angeles 90033, CA, USA; ^3^Keck School of Medicine of USC, Los Angeles 90033, CA, USA

## Abstract

**Background:**

Hemodynamic-guided therapy using the CardioMEMS™ system has been shown to reduce heart failure hospitalization (HFH) in both clinical trials and real-world settings. However, the CardioMEMS system requires input from multiple independent elements to achieve its effect, and no studies have been done to investigate those elements. Consistent patient participation and health care provider participation are two of those key elements, and this study sought to assess how they affect HFHs.

**Methods:**

This was a single-center, retrospective cohort study of patients with the CardioMEMS sensor. The primary outcome was the number of HFH days patients experienced in the 1 year following CardioMEMS sensor implant. The primary independent variables were the average number of days between patient transmissions of data and the average number of days between health care provider reviews of those data. Covariates included patient demographics, medical comorbidities, history of HFHs, and initial pressure response to hemodynamic-guided therapy at 28 days after implant. Data were fit to a zero-inflated negative binomial regression.

**Results:**

Seventy-eight patients were included in the study. The mean age was 64 ± 15 years, 52 (67%) were male, and 58 (76%) had heart failure with reduced ejection fraction. During the study period, there were 538 cumulative HFH patient-days. Based on the regression model, there was an exponential relationship between HFH days and the mean number of days between patient transmissions (IRR = 1.74, 95% CI: 1.09–2.75, *p*=0.019). There was also an exponential relationship between HFH days and the mean number of days between health care provider reviews (IRR = 1.03, 95% CI: 1.01–1.05, *p*=0.013).

**Conclusions:**

This single-center study suggests that more frequent patient transmissions and health care provider reviews of the CardioMEMS system are associated with a decreased number of HFH days, but larger multicentered studies are required. Further systems-based analyses of the CardioMEMS system may be a useful approach to guiding effective use of the CardioMEMS device.

## 1. Introduction

Heart failure (HF) is a leading cause of morbidity and mortality in the United States, mentioned in 1 out of 9 death certificates in 2011, and designated as the underlying cause in 58,309 out of 284,388 deaths [1]. The associated financial burden is massive and expected to double to more than $70 billion in the United States by 2030 [2, 3]. Reducing heart failure hospitalizations (HFHs) and decreasing associated morbidity and mortality remain key priorities of management. New strategies for meeting these goals are a constant focus of heart failure research [4–7].

The CardioMEMS™ HF system includes an implantable pulmonary artery pressure (PAP) sensor that was approved for use by the Food and Drug Agency (FDA) in 2014 for New York Heart Association (NYHA) functional class III patients with a prior HFH within the preceding 12 months. The CHAMPION (CardioMEMS Heart Sensor Allows Monitoring of Pressure to Improve Outcomes in NYHA Class III Heart Failure Patients) trial, open-access registry, and several subgroup analyses subsequently confirmed a reduction in HFH in patients using this sensor [8–11].

**Figure 1:**
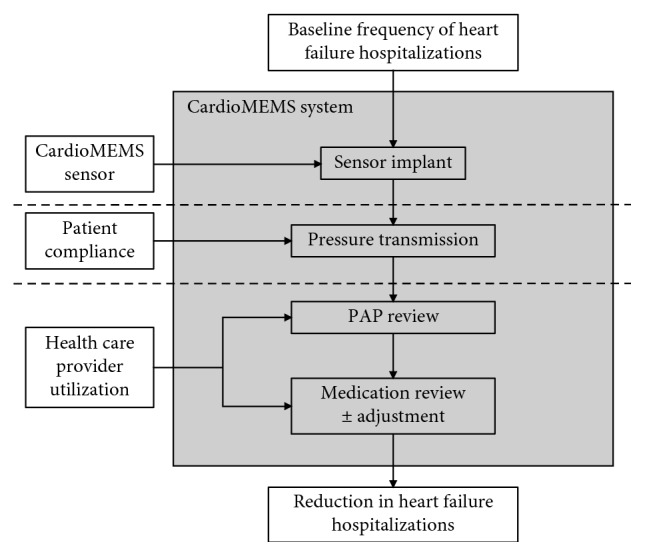
The CardioMEMS system. It requires multiple inputs to achieve its aim of reducing heart failure hospitalizations. The sensor must be implanted correctly and safely, patients must transmit pulmonary artery pressure (PAP) data regularly, and health care providers must review those data and formulate treatment plans. Patients can transmit their PAP data at home or in other nonhospitalized settings, and health care providers review data in an independent process at a separate time interval.

However, effectiveness of the CardioMEMS™ sensor requires not only safe sensor implantation but also appropriate stewardship by both patients and health care providers. Patients must upload their pulmonary artery pressure (PAP) data on a regular basis, and health care providers must subsequently review these data and react accordingly, typically through titration of medical therapy (Figure 1).

Patients initiate the transmission of their pressure data by placing a handheld “wand” near their chests. This wand communicates with the intra-arterial pressure sensor, extracting PAP data at that time point and uploading them to Merlin.net, a secure online database. In a separate process, health care providers review uploaded pressure data and react by changing medication dosing or changing the timing of clinic follow-ups. If pressure data are not available, such as when a patient forgets to transmit data, health care providers can send reminders to patients to do so. Providers document their review of pressure data and their plans going forward on the Merlin.net website. To date, no study has taken a systems-based approach to understand the impact of the patient-specific and health care provider-specific uses of the CardioMEMS™ HF system on HFH.

The purpose of this study was to apply a systems-based approach to examine whether the frequency of patient pressure transmissions and the frequency of health care provider reviews of those data were associated with risk of HFH.

## 2. Methods

This was a single-center, retrospective cohort study of patients who received the CardioMEMS™ pressure sensor at Keck Medical Center of the University of Southern California from October 2014 to August 2017. Patients were included if they had had the sensor for at least 12 months at the time of data collection and regardless of left ventricular ejection fraction. Patients with a left ventricular assist device were included. This study was approved by the Institutional Review Board at the University of Southern California by waiver of consent.

A systems' context diagram was constructed to reflect the key inputs affecting HFH within the CardioMEMS system and served as a guide for data collection. Demographics, medical comorbidities, and the number of HFH days in the one year prior to CardioMEMS implant were obtained through the chart review. These data were handled as covariates in statistical modeling. The frequency of patient pressure transmissions and the frequency of health care provider reviews were the key independent variables. These data were collected from the Merlin.net website. Patient pressure transmission was defined as receipt and documentation of that pressure on the Merlin.net website. A health care provider review was defined as a note on the Merlin.net website that documented data reviews and a treatment plan, which could include reminding a patient to transmit data.

The frequency of patient pressure transmissions was characterized in two ways by (1) the mean number of days between pressure transmissions and (2) the number of times patients did not transmit pressures for more than 7 days during one year following CardioMEMS sensor implantation. The frequency of health care provider reviews was characterized by (1) the mean number of days between health care provider reviews and (2) the number of times there was no review of pressure data for more than 7 days. A secondary hypothesis was that the patient's initial pressure response to the remote hemodynamic monitoring management strategy would predict future HFH. To explore this, a pressure-response variable was constructed and defined as the difference in mean PA diastolic pressure between the first and second two weeks following sensor implantation. This pressure-response variable was an additional independent variable explored in our statistical model.

The primary outcome was the total number of days a patient spent hospitalized for heart failure in the 1 year following CardioMEMS™ implant. HFH data were obtained from an individual patient chart review. HFH was defined as any hospitalization with reason for admission being directly related to an acute heart failure exacerbation. Each case was reviewed by two independent physicians (JST and AMW), who adjudicated the cause of hospitalization and determined whether or not the primary reason for hospitalization was due to HF.

Baseline clinical characteristics, patient compliance with transmission, health care provider review, and hospitalization statistics were calculated and presented as percent when classified categorically, mean/standard deviation when normally distributed, and quartiles when nonnormally distributed. Initial descriptive analysis of outcome data revealed a high number of patients with a zero count of days spent in HFH and wide distribution of nonzero count data, so data were fit to a zero-inflated negative binomial (ZINB) regression. The initial binomial component identified characteristics of patients not likely to have spent any days in HFHs, and the subsequent negative binomial component assessed factors predictive of the count of days spent in HFH, drawn from the subgroup of patients determined to be at risk for an HFH event based on the initial binomial component. The ZINB model was constructed in a stepwise elimination fashion. A *p* value <0.25 on univariate analysis was required for inclusion in the initial model. A *p* value ≤0.05 was deemed statistically significant in the final model. The model's constant term, which is the hypothetical value of the outcome variable if all continuous independent variables could be set to zero and all categorical independent variables were presumed to be the reference groups, was reported to facilitate risk projections. Post hoc validation was done through examination of Pearson residual distribution and leverage plots. The ZINB model was then used to project the number of HFH days patients might spend in the year following sensor implant based on a patient's stewardship and a health care provider's stewardship of the CardioMEMS sensor. STATA 14.2 was used for all data management and statistical analyses.

## 3. Results

Between October 2014 and August 2017, 105 patients received a CardioMEMS™ sensor, and 78 patients met criteria for inclusion. Twenty-six patients were excluded because they had less than one year of pressure data, and one patient with congenital heart disease was excluded. Baseline patient characteristics are shown in Table 1. Notably, there were twenty patients (24%) with a left ventricular ejection fraction (LVEF) >40%, and of patients with LVEF ≤ 40%, the median LVEF was 25%. Forty-seven patients (59%) showed a reduction in mean pulmonary artery (PA) diastolic pressure by the fourth week following sensor implant (pressure response). In patients with a pressure response, the average change in PA diastolic pressure was −2.91 ± 2.69 mm·Hg. In patients without a pressure response, the average change in PA diastolic pressure was 3.13 ± 2.44 mm·Hg. The pressure response for all patients was −0.49 ± 3.96 mm·Hg. Patients transmitted data a median of 1.5 days apart. Health care provider reviews occurred at a median of 6.3 days apart (Table 2).

The 78 patients in this study spent a total of 538 patient-days hospitalized for HF in the 1 year after CardioMEMS™ implant. Fifty-three patients did not spend any time in HFH, and the remaining 25 patients demonstrated a wide distribution of the number of days spent in HFH (alpha coefficient = 0.75, likelihood ratio ${\bar{\chi }}^{2}\left(01\right)=221.6,\;p<0.001$) (Table 3).

**Figure 2:**
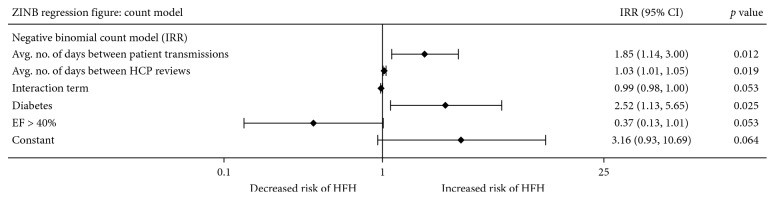
Forest plot illustration of the count model component of the zero-inflated negative binomial model. Regression coefficients and 95% confidence intervals are presented as incidence rate ratios. The interaction term is defined as the product of the average number of days between patient transmissions and the average number of days between health care provider reviews; it captures the synergistic impact of a patient's stewardship and a health care provider's stewardship of the CardioMEMS sensor. The constant term indicates the *y*-intercept of this model and can be interpreted as the number of days patients are predicted to spend in HFH if the value of all the independent variables could be set to zero. ZINB: zero-inflated negative binomial; IRR: incidence rate ratio; CI: confidence interval; Avg. no.: average number; EF: ejection fraction; HFH: heart failure hospitalization.

The negative binomial count component of the ZINB (Figure 2) revealed an exponential relationship between the number of days patients spent in HFHs and the mean number of days between their PAP transmissions (incidence rate ratio (IRR) = 1.85, 95% confidence interval (CI): 1.14–3.00, *p*=0.012). Similarly, there was an exponential relationship between the number of days patients spent in HFHs and the mean number of days between health care provider reviews (IRR = 1.03, 95% CI: 1.01–1.05, *p*=0.019). The interaction term between patient transmissions and health care provider reviews suggested an inverse relationship with the number of days spent in HFH but did not meet statistical significance (*p*=0.053). Diabetes was a risk factor for spending a greater number of days in HFH, and this relationship was statistically significant (IRR = 2.52, 95% CI: 1.13–5.64, *p*=0.025). Ejection fraction >40% was correlated with spending fewer days in HFH, but this did not meet statistical significance (*p*=0.053).

**Figure 3:**
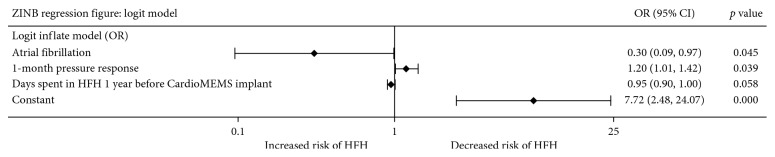
Forest plot illustration of the logit model component of the zero-inflated negative binomial model. Regression coefficients and 95% confidence intervals are presented as odds ratios. The 1-month pressure response is defined as the difference in mean pulmonary artery diastolic pressure between the first and second two weeks following sensor implantation (${\bar{\text{PADP}}}_{\text{D}1⟶\text{D}14}-{\bar{\text{PADP}}}_{\text{D}15⟶\text{D}28}$). Of note, an odds ratio (OR) >1 indicates increased probability of having zero HFHs, and an OR <1 indicates increased probability of having HFHs. The constant term indicates the *y*-intercept of this model and can be interpreted as the odds of having zero days spent in HFH if the value of all the independent variables could be set to zero. ZINB: zero-inflated negative binomial; OR: odds ratio; CI: confidence interval; HFH: heart failure hospitalization.

The inflate component of the ZINB model (Figure 3) identifies characteristics of patients not likely to spend any days in HFHs; thus, an odds ratio (OR) >1 was protective against HFHs, and an OR <1 signified increased risk for HFHs. The initial pressure response to CardioMEMS™-directed therapy was found to be protective against HFHs (OR = 1.20, 95% CI: 1.01–1.42, *p*=0.039), while having a history of atrial fibrillation was found to be a risk factor for HFHs (OR = 0.30, 95% CI: 0.09–0.97, *p*=0.045). Spending a larger number of days hospitalized for heart failure 1 year prior to CardioMEMS implant was correlated with an increased risk for HFH (OR = 0.95, 95% CI: 0.90–1.00, *p*=0.058) but did not meet statistical significance.

**Figure 4:**
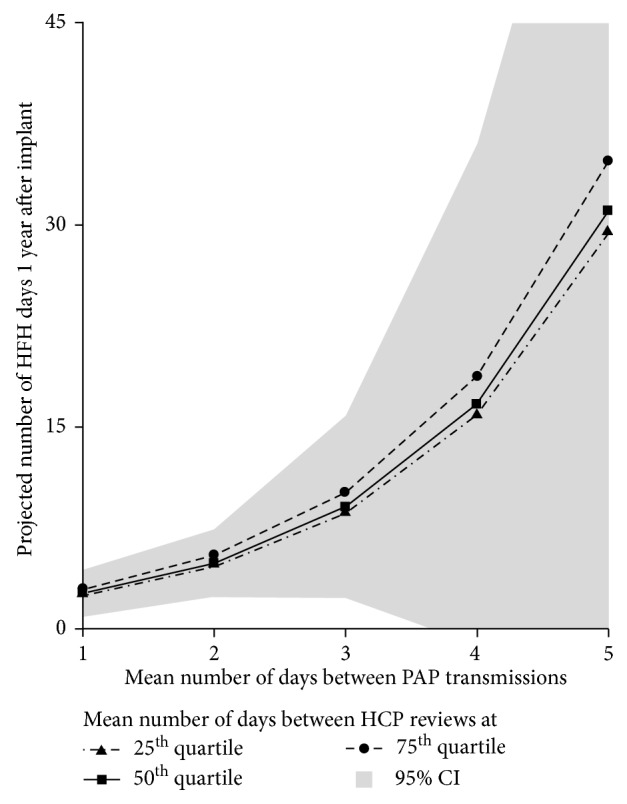
Heart failure hospitalization projections from the zero-inflated negative binomial model. Using these projections, this figure illustrates the exponential relationship between the number of HFH days a patient is projected to spend in the 1 year after sensor implant, based on the frequency of that patient's transmissions of PAP data. Three separate projections are presented to demonstrate the impact of health care provider reviews. These projections were calculated at the 25^th^/50^th^/75^th^ quartiles of health care provider reviews of PAP data, which correspond to health care provider reviews at a mean of 4.4/6.3/10.3 days, respectively. The gray shaded area illustrates the 95% confidence interval for the projection made at the 50^th^ quartile of the health care provider review. HFH: heart failure hospitalization; PAP: pulmonary artery pressure; HCP: health care provider; CI: confidence interval.

Results of the ZINB model projections can be found in Figure 4. Of note, the projections assume patients do not have diabetes and have an LVEF ≤40%. As an example of these projections, patients who transmit PAP data an average of 4 days apart spend an additional 14 days in the hospital in the first year after CardioMEMS implant, compared to their peers who transmit pressures everyday. Similarly, a health care provider who reviews data every 10^th^ day vs. every 4^th^ day costs a patient between 1 and 7 days in HFH days depending on how often that patient transmits PAP data.

## 4. Discussion

The main finding of our study of patients receiving hemodynamic-guided therapy was that patient and provider utilization of the CardioMEMS™ HF system was associated with risk of future HFH. Patients who transmit PAP data more frequently appear to spend less time hospitalized for HF. Similarly, patients whose health care providers review PAP data more frequently appear to spend fewer days in HFH. Although these findings seem intuitive, this is the first study to implement a systems-based approach to quantify the impact that patients and health care providers can have on future HFH.

Model projections were presented to demonstrate the ease and feasibility of translating our statistical results into clinical outcomes. The projections directly translate patients and health care providers' daily actions into days patients may spend hospitalized for HF, which is a much more tangible outcome compared to a regression coefficient. The projections presented in this study are not intended to provide estimates for a generalized population but rather to serve as a proof of concept.

Other nonmodifiable risk factors associated with an increase in the number of days spent in HFH were HF with reduced ejection fraction and a history of diabetes mellitus. There was a suggestion that LVEF >40% was associated with spending fewer days in HFH (IRR = 0.37, 95% CI: 0.13–1.01, *p*=0.053). These trends are consistent with the original results of the CHAMPION trial, where patients with LVEF >40% demonstrated a slightly greater benefit from the CardioMEMS sensor compared to those with LVEF <40% (absolute risk reduction (ARR) of 17% in the LVEF > 40% population vs. ARR of 11% in the LVEF ≤ 40% population) [10]. Our finding that diabetes mellitus increases a patient's risk for HFH is in line with prior studies [12]. Whether or not more frequent transmission and review practices in diabetic patients and/or those with LVEF <40% can further improve outcomes is intriguing but beyond the scope of our study design.

It should be noted that the interaction term between patient transmissions and health care provider reviews suggested a protective effect against HFH, although the term failed to meet statistical significance (IRR = 0.988, *p*=0.053). One interpretation of this finding is that patients who transmit infrequently with infrequent reviews by their health care providers spend fewer days in HFH. While the term was not statistically significant, the term's relevance to the model may have been driven by a unique subset of patients that were previously undermedicated and after initial sensor implant and medication titration and optimization, no longer required close observation by a patient or provider.

Our results also suggest there is a group of patients who are at low risk of HFH after sensor implant, largely independent of patient and health care provider use of the sensor. The zero-inflated logit aspect of the ZINB is classically used to define a phenotype of subjects not at risk of the outcome of interest and filter those subjects out of the count model aspect of the ZINB, thereby increasing sensitivity of the overall model. While all patients in this study had been clinically identified to be at risk for HFHs, our model did identify a low-risk phenotype associated with the patients who did not have any HFHs. This low-risk phenotype patient did not have atrial fibrillation and exhibited a pressure response.

The characteristics associated with low risk for HFH identified in this study are consistent with results of previous studies. Pressure data analysis of the COMPASS-HF trial, which studied the Medtronic Chronicle implantable hemodynamic monitoring sensor, revealed prognostic implications of a low average pulmonary artery diastolic pressure (PADP). These analyses showed that patients with no HFH events had a significantly lower average PADP compared to patients with one or more HFH events [13, 14]. Atrial fibrillation has long been shown to contribute to HF morbidity in both large meta-analyses and clinical trials [15, 16], and recent trials have shown that the termination of atrial fibrillation through catheter ablation results in decreased mortality [17, 18]. Overall, these studies in conjunction with our own suggest that patients with atrial fibrillation and with elevated PADP despite CardioMEMS-sensor-directed therapies remain at elevated risk of HFHs and require close observation by patients and providers.

## 5. Limitations

Our study has several limitations: First, the overall study cohort is relatively small with all patients enrolled from a single academic institution. Second, variability of health care provider response to patient pressure trends was not captured nor implemented by our model; each provider had their own unique strategy for patient management. Future multicenter studies with a larger sample size will be necessary to more definitively quantify the impact that patients and health care providers have on future HFH, as well as a more refined patient phenotyping for patients that may or may not respond favorably to a remote hemodynamic monitoring strategy.

## 6. Conclusions

A systems-based analysis of patients managed with a remote hemodynamic monitoring strategy may be a useful approach for isolating specific factors that enhance efficacy of a remote hemodynamic monitoring management strategy.

## Figures and Tables

**Table 1:** Baseline descriptive characteristics.

*Demographic information*	
Age	64.4 [14.8]
Male	52 (66.7%)
White	42 (53.8%)
Left ventricular ejection fraction >40%	20 (24.1%)
Ischemic cardiomyopathy	36 (46.2%)
Implantable cardiac defibrillator	51 (65.4%)
Left ventricular assist device	11 (14.1%)
*NYHA class*	
III	76 (97.4%)
IV	2 (2.6%)

*Medical comorbidities*	
Hypertension	47 (60.2%)
Coronary artery disease	38 (48.7%)
Diabetes mellitus	38 (48.7%)
Atrial fibrillation	46 (59.0%)
Chronic obstructive pulmonary disease	8 (10.3%)
Chronic kidney disease stage IV or V	13 (16.7%)

*Medications* ^*∗*^	
Beta-blocker	69 (88.5%)
ACE inhibitor/ARB	32 (41.0%)
Aldosterone antagonist	34 (43.6%)
Loop diuretic	67 (85.9%)
Angiotensin receptor-neprilysin inhibitor	6 (7.7%)
Organic nitrate	13 (16.7%)
Hydralazine	19 (24.4%)
Inotrope (home infusion)	8 (10.3%)

*Hemodynamic analyses* ^*∗*^	
Systolic blood pressure	114 [17.6]
Diastolic blood pressure	64 [11.5]
Heart rate	76 [11.9]
Baseline pulmonary artery diastolic pressure	24.7 [7.9]

Note: categorical data are presented as number (percent), and continuous data are presented as mean [standard deviation]. ^*∗*^Data obtained at time of CardioMEMS sensor implant. NYHA: New York Heart Association; ACE: angiotensin-converting enzyme; ARB: angiotensin receptor blocker.

**Table 2:** Patient and health care provider's sensor utilization practices.

*Patient transmission practices*	
Mean number of days between transmissions	1.1/1.5/2.3
Count where time between transmissions >7 days	0/2/4

*Health care provider review practices*	
Mean number of days between reviews	4.4/6.3/10.3
Count where time between reviews >7 days	6/14/20

Note: continuous data are presented as 25^th^/50^th^/75^th^ quartiles, as data were not normally distributed; count data are presented as 25^th^/50^th^/75^th^ quartiles.

**Table 3:** Heart failure hospitalization statistics.

*Outcome descriptors*	
Number of patients with a zero count of HFH	53 (67.9%)
Days spent in HFH out of the first year with CardioMEMS™ sensor (all patients)	0/0/5
Days spent in HFH out of the first year with CardioMEMS™ sensor (nonzero count patients)	7/11/30

*Pre-CardioMEMS implant hospitalization rate*	
Number of days spent in HFH 1 year before CardioMEMS™	0/7/12
Number of days spent hospitalized for any cause 1 year before CardioMEMS™	4/13/28

Note: count data are presented as 25^th^/50^th^/75^th^ quartiles, categorical data are presented as number (percent).

## Data Availability

The patient data used to support the findings of this study are restricted by the USC Institutional Review Board in order to protect patient privacy. Data are available from the corresponding author (jeffrest@med.usc.edu) for researchers who meet the criteria for access to confidential data.
